# Techniques of Fluorescence Cholangiography During Laparoscopic Cholecystectomy for Better Delineation of the Bile Duct Anatomy

**DOI:** 10.1097/MD.0000000000001005

**Published:** 2015-06-26

**Authors:** Yoshiharu Kono, Takeaki Ishizawa, Keigo Tani, Nobuhiro Harada, Junichi Kaneko, Akio Saiura, Yasutsugu Bandai, Norihiro Kokudo

**Affiliations:** From Hepato-Biliary-Pancreatic Surgery Division, Department of Surgery, Graduate School of Medicine, the University of Tokyo, Tokyo, Japan (YK, TI, KT, NH, JK, NK); Department of Gastroenterological Surgery, Cancer Institute Hospital, Japanese Foundation for Cancer Research, Tokyo, Japan (TI, AS); and Department of Surgery, Tokyo Yamate Medical Center, Japan Community Health Care Organization, Tokyo, Japan (NH, YB).

## Abstract

To evaluate the clinical and technical factors affecting the ability of fluorescence cholangiography (FC) using indocyanine green (ICG) to delineate the bile duct anatomy during laparoscopic cholecystectomy (LC).

Application of FC during LC began after laparoscopic fluorescence imaging systems became commercially available.

In 108 patients undergoing LC, FC was performed by preoperative intravenous injection of ICG (2.5 mg) during dissection of Calot's triangle, and clinical factors affecting the ability of FC to delineate the extrahepatic bile ducts were evaluated. Equipment-related factors associated with bile duct detectability were also assessed among 5 laparoscopic systems and 1 open fluorescence imaging system in ex vivo studies.

FC delineated the confluence between the cystic duct and common hepatic duct (CyD–CHD) before and after dissection of Calot's triangle in 80 patients (74%) and 99 patients (92%), respectively. The interval between ICG injection and FC before dissection of Calot's triangle was significantly longer in the 80 patients in whom the CyD–CHD confluence was detected by fluorescence imaging before dissection (median, 90 min; range, 15–165 min) than in the remaining 28 patients in whom the confluence was undetectable (median, 47 min; range, 21–205 min; *P* < 0.01). The signal contrast on the fluorescence images of the bile duct samples was significantly different among the laparoscopic imaging systems and tended to decrease more steeply than those of the open imaging system as the target-laparoscope distance increased and porcine tissues covering the samples became thicker.

FC is a simple navigation tool for obtaining a biliary roadmap to reach the “critical view of safety” during LC. Key factors for better bile duct identification by FC are administration of ICG as far in advance as possible before surgery, sufficient extension of connective tissues around the bile ducts, and placement of the tip of laparoscope close and vertically to Calot's triangle.

## Abstract 2

Supplemental Digital Content is available in the text

## INTRODUCTION

Since radiographic intraoperative cholangiography (IOC) was first reported in 1931,^1^ this procedure has been widely used as almost the only method by which to delineate the bile duct anatomy during cholecystectomy. Several studies have revealed that routine use of IOC during laparoscopic cholecystectomy (LC) may reduce the incidence of bile duct injury,^2,3^ or at least its severity.^4,5^ In practice, however, the frequency of use of IOC during LC varies widely among surgeons and hospitals, probably because of several disadvantages of IOC: a longer operation time, the need for additional medical resources, exposure of the patient and medical staff to radiation, and an increased risk of bile duct injury caused by insertion of a transcystic tube.^6^ Intraoperative ultrasonography is also an established modality for confirming the bile duct anatomy and presence of gallstones during LC^3^; however, it requires considerable skill to scan the biliary tract and interpret cross-sectional ultrasonographic images.^7^

A novel IOC technique that utilizes the fluorescence of indocyanine green (ICG) excreted in the bile after intravenous injection was recently developed for use in open surgery.^8^ Later, as laparoscopic/robotic fluorescence imaging systems became commercially available, this technique began to be applied in the clinical setting during laparoscopic/robotic cholecystectomy worldwide, including in Japan,^9,10^ the United States,^11,12^ Europe, ^13–15^ and Argentina.^16^ These previous series, including our own, have suggested the potential advantage of fluorescence cholangiography (FC) with respect to its ability to enable real-time identification of the extrahepatic bile ducts during dissection of Calot's triangle, without exposing either the patient or the surgical team to irradiation. However, the clinical and technical factors that may affect the ability of FC to delineate the bile duct anatomy remain unclear because of the insufficient size of the study population and lack of comparative data on the bile duct detectability among fluorescence imaging systems. The aim of this study was to demonstrate the optimal conditions and technical details of FC that will allow it to be used widely and efficiently as an intraoperative navigation tool of the bile duct anatomy during LC.

## METHODS

This observational study was conducted with the approval of the institutional ethics committees and registered in the UMIN Clinical Trials Registry (registration number UMIN000001075; http://www.umin.ac.jp/ctr/index.htm). Informed consent was obtained from all of the patients.

### Principle of ICG-Fluorescence Imaging

ICG binds to plasma proteins. When illuminated with near-infrared light, the protein-bound ICG emits light with a peak wavelength of about 830 nm.^17^ We hypothesized that fluorescence imaging following intravenous injection of ICG could provide images of the biliary tract because ICG is excreted exclusively into the bile and human bile contains plasma proteins that bind with ICG. Biliary excretion of ICG starts within minutes after intravenous injection, peaking within 2 hours and continuing for as long as 20 hours.^18^

### Fluorescence Cholangiography Techniques During LC

First, 1 mL of ICG (2.5 mg/mL; Daiichi Sankyo Co., Tokyo, Japan) is intravenously injected prior to the surgery. When ICG is administered during surgery, fluorescence images of the bile ducts should be obtained at least 15 minutes after the intravenous injection so that the ICG is sufficiently washed out from the connective tissues around the bile ducts. The fluorescence in the bile ducts can persist for more than 7 hours following the administration of ICG.^9^

**FIGURE 1:**
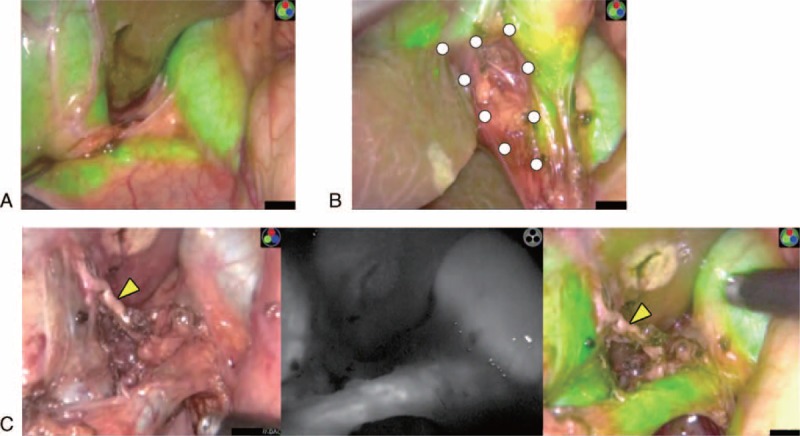
Surgical techniques of FC during LC. (A) FC prior to dissection of Calot's triangle. Fluorescence images of the CyD, CHD, and gallbladder are indicated in green in the full-color images. (B) FC from the dorsal aspects of Calot's triangle (dotted line) indicates that no fluorescence structures consistent with the accessory hepatic ducts exist in this layer of the dissection. (C) Full-color image (left), monochromatic FC image (middle), and fusion FC image (right) after dissection of Calot's triangle. Arrowheads show the cystic artery. All images were obtained using LS5. See Video, Supplementary Video 1, http://links.lww.com/MD/A307.

After dissecting the adhesions around the hepatoduodenal ligament, FC is performed to identify the anatomy of the extrahepatic bile ducts by changing the full-color images to fluorescence images using filter switches on the camera head and/or light source in the laparoscopic imaging system (Figure 1A). Calot's triangle is then dissected to reach the “critical view of safety,”^19^ using FC to confirm the presence or absence of the accessory bile ducts at any time during the procedures. Fluorescence images of the bile ducts should be obtained from both the ventral and dorsal aspects of Calot's triangle (Figure 1B and C, see Video, Supplementary Video 1, http://links.lww.com/MD/A307 [**Supplementary Video 1.** Techniques of FC during dissection of Calot's triangle in LC, 3 minutes 48 seconds, 97MB], which demonstrates techniques of FC during LC). Finally, the cystic duct (CyD) is closed and then divided after confirming that no fluorescing structures exist with the exception of the CyD between the gallbladder and the common hepatic duct (CHD).

### Evaluation 1: Clinical Factors Affecting Bile Duct Detectability by FC

#### Patients

The study subjects comprised 108 patients who underwent LC from July 2008 to October 2012 at the Tokyo Yamate Medical Center (Tokyo, Japan) or The University of Tokyo Hospital. Thirteen patients were excluded from the study because the fluorescence imaging system was unavailable on the day of surgery. A concomitant diagnosis of common bile duct (CBD) stones was made in 6 patients, who underwent endoscopic retrograde cholangiography performed prior to LC (n = 5) or by simultaneous laparoscopic choledocholithotomy (n = 1). Laparoscopic gastric wedge resection was performed in one patient, and lymph node dissection to treat gallbladder cancer was performed following LC in another patient.

The patients comprised 49 men and 59 women with a median age of 56 years (range, 19–82 years). The median body mass index (BMI) was 23.5 kg/m^2^ (range, 15.6–42.2 kg/m^2^). Six patients were preoperatively diagnosed with acute cholecystitis according to the Tokyo Guidelines.^20^ All patients underwent preoperative cholangiography, including magnetic resonance cholangiopancreatography (n = 75), drip infusion cholangiography^21^ or drip infusion cholangiography–computed tomography (n = 47), and/or endoscopic retrograde cholangiography (n = 7) as a routine preoperative evaluation before LC at both institutions.

### Evaluation of the Ability of FC to Delineate the Bile Duct

FC was performed during LC using the prototype imaging system (described as LS1 in *Evaluation 2*) following intravenous injection of ICG (2.5 mg) either prior to the entry of the patient into the operation room (n = 43) or just after intratracheal intubation in the operation room (n = 65).

The ability of FC to delineate the bile duct anatomy was evaluated based on criteria images by the operating surgeon, using the confluence between the CyD and CHD as a landmark anatomical structure for the evaluation. Clinical factors that could affect the bile duct detectability by FC were analyzed.

### Evaluation 2: Equipment-Related Factors Affecting Bile Duct Detectability by FC

#### Fluorescence Imaging Systems

Five laparoscopic fluorescence systems (LS1–5) were used in this study (Table 1); LS1 was the prototype fluorescence imaging system (Hamamatsu Photonics, Hamamatsu, Japan and Shinko Optical, Tokyo, Japan) that was used in all patients included in *Evaluation 1*. LS2 is a standard-definition fluorescence imaging system for use in clinical research (Olympus Medical Systems, Tokyo, Japan). LS3 is a high-definition model of LS1 that has been commercially available in Japan since 2014. LS4 is a commercially available high-definition fluorescence imaging system (Karl Storz, Tuttlingen, Germany). In the present study, fluorescence images were obtained under image processing similar to narrow-band imaging (spectra A mode) to enhance the contrast of the fluorescence signals. LS5 is also a commercially available high-definition imaging system (Novadaq, Toronto, Canada), and enables superimposition of fluorescence images on full-color images. Before creating fusion images on color images, monochromatic images were used to calculate the fluorescence intensities (FIs) in this study. A conventional fluorescence imaging system for open surgery (OS1; Hamamatsu Photonics) was used for reference.

**TABLE 1:**
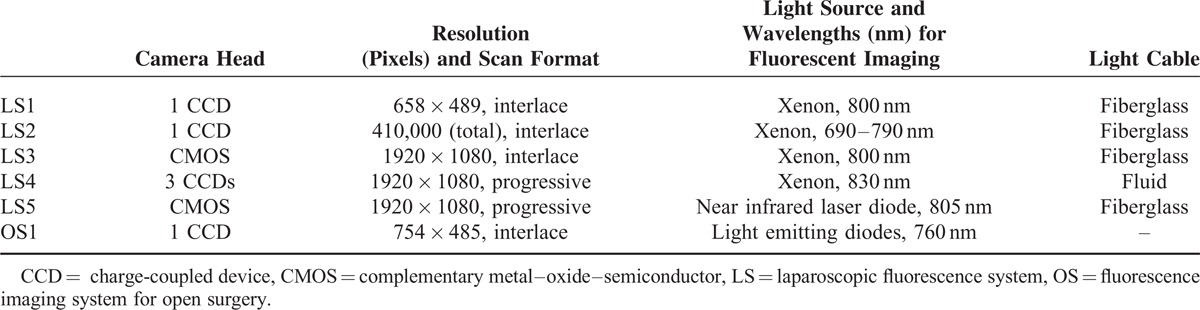
Specifications of Fluorescent Imaging Systems

### In Vitro Evaluation of Equipment Differences in Bile Duct Detectability

First, the sensitivity of the fluorescence signals dependent on the concentrations of ICG was evaluated. Fluorescence signals of a phantom containing 5 resins made of ICG diluted with alcohol at a dose of 0.5 (0.00039 mg/mL), 1.0, 2.0, 5.0, and 20.0 μmol/L were obtained using fluorescence imaging systems (LS1–5 and OS1). The tip of the laparoscope or camera head was set 10 cm above the phantom in a dark box.

Second, the distance-dependent sensitivity of the fluorescence imaging systems was evaluated. Bile duct samples were made by injecting human bile juice mixed with ICG at a dose of 0.025 mg/mL into a 4-mm plastic tube. Fluorescence images of the bile juice samples were then obtained by setting the tip of the laparoscope at 5, 10, and 15 cm above the samples.

Finally, the tissue permeability of the fluorescence imaging systems was estimated by obtaining fluorescence signals emitted from the bile juice samples covered with 1, 2, 3, 4, or 5 slices of pork (thickness of approximately 1.5 mm) from 10 cm above the sample.

Still images were obtained 10 times during each observation. The FIs in the regions of interest, which were set in the fluorescence regions and in the background, were then calculated with a range of 0–255 using Photoshop CS5 software (Adobe Systems, San Jose, CA). Signal contrast was calculated using the following formula:

Signal contrast = (FI in fluorescence regions– FI in background)/255

### Statistical Analysis

Categorical data and continuous data were compared using Fisher's exact test and Wilcoxon's rank sum test, respectively. Multiple comparisons were performed using the Steel–Dwass method. A *P*-value of <0.05 was considered to denote statistical significance. Statistical analysis was performed with JMP software (version 9.0.2; SAS Institute, Inc., Cary, NC).

## RESULTS

### Evaluation 1: Clinical Factors Affecting Bile Duct Detectability by FC

#### Ability of FC to Delineate the Bile Duct Anatomy

**FIGURE 2:**
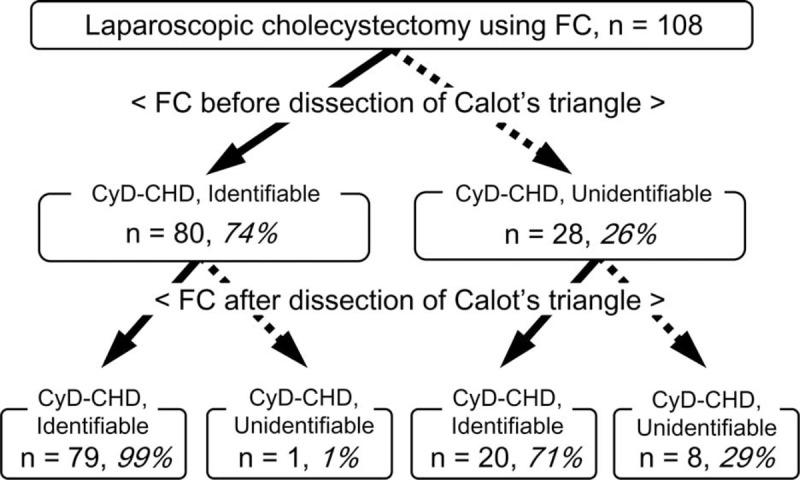
Flow diagram of the results of FC before and after dissecting Calot's triangle. The CyD–CHD confluence could be visualized by FC before dissecting Calot's triangle in approximately 75% of the patients; the CyD–CHD confluence could also be visualized by FC after dissecting Calot's triangle in all the patients, except for 1 in whom the ICG spilled out from the gallbladder during the procedures (a). Among the remaining 25% of patients in whom FC failed to delineate the CyD–CHD confluence before dissecting Calot's triangle, the confluence could be identified after the dissection in approximately 70% of patients. In 8 patients including 1 case of acute cholecystitis (b), FC failed to delineate the CyD–CHD confluence even after dissecting Calot's triangle.

Table 2 and Figure 2 summarize the ability of FC to delineate the bile duct anatomy using the prototype imaging system (LS1). The success rates for identifying the CyD–CHD confluence by FC before and after dissecting Calot's triangle were 74% and 92%, respectively. When the CyD–CHD confluence was identified by FC before dissecting Calot's triangle, the confluence was also identifiable by FC after dissecting Calot's triangle in all patients but one, in whom the gallbladder wall was opened during the dissection and bile juice containing ICG spilled out. In contrast, FC after dissecting Calot's triangle enabled identification of the CyD–CHD confluence in 20 of the 28 patients (71%) in whom it was unidentifiable by FC performed before dissecting Calot's triangle. In the remaining 8 patients, FC failed to delineate the CyD–CHD confluence even after dissecting Calot's triangle. The BMIs of these patients ranged from 16.6 to 40.8 kg/m^2^ (median, 27.4 kg/m^2^), and in 1 patient, LC was performed for acute cholecystitis.

**TABLE 2:**
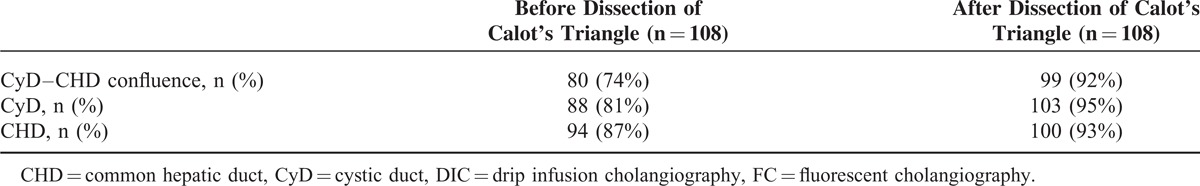
The Ability of FC to Delineate the Bile Duct Anatomy

**FIGURE 3:**
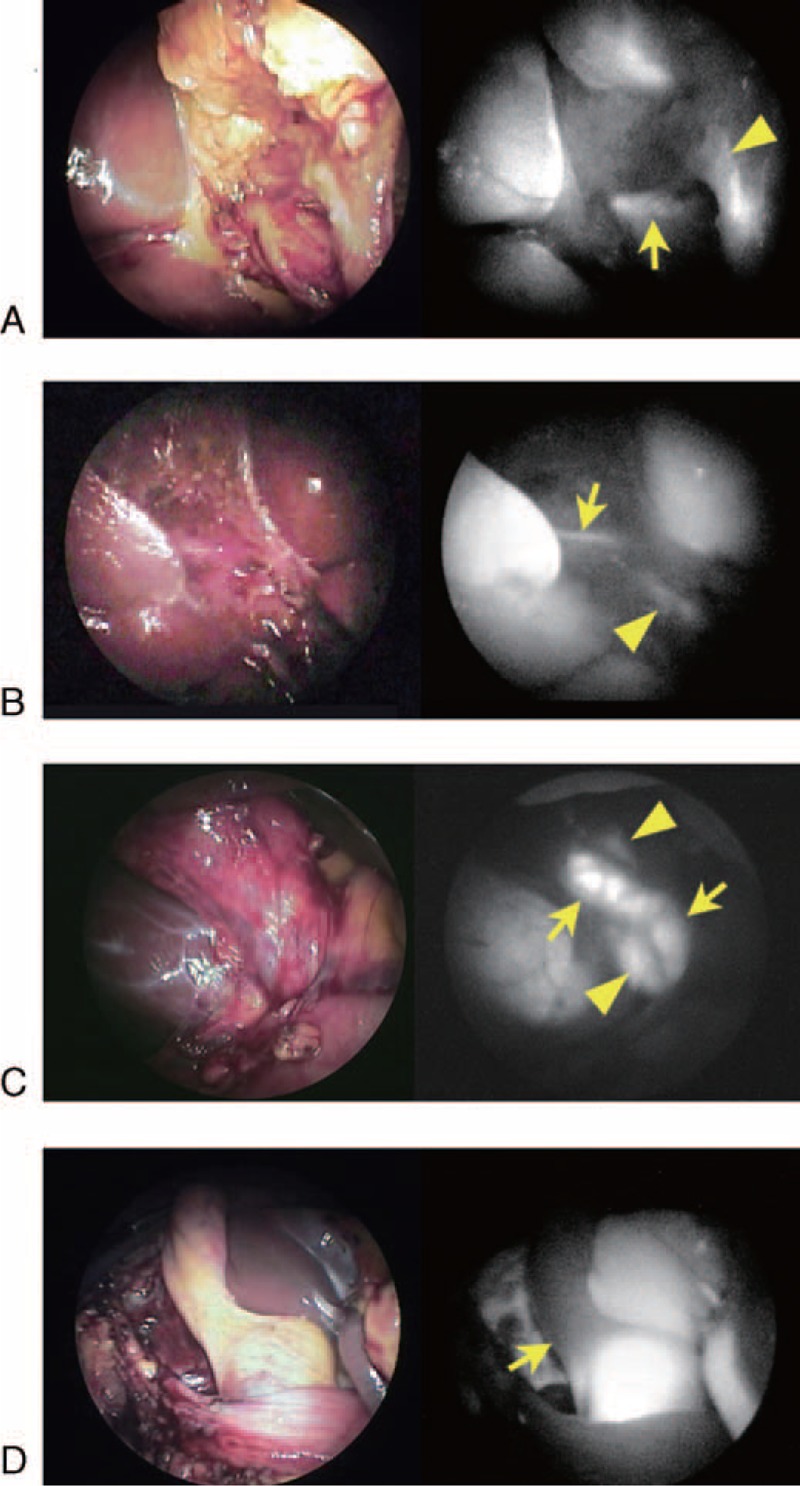
Additional findings obtained by FC. (A) The accessory hepatic duct (arrow) draining the right lateral sector could be visualized by FC after dissecting Calot's triangle. This duct ran caudally along Rouviere's sulcus and drained directly into the CHD. The arrowhead indicates the CyD. (B) A thin hepatic duct running along the gallbladder bed and probably draining Couinaud's segment V (arrow) was visualized by FC after dissecting Calot's triangle. The arrowhead indicates the CHD. (C) The CyD (arrows) could be visualized by FC before the dissection of Calot's triangle, running anteriorly and draining into the CHD (arrowheads) from its left side. (D) FC before dissecting Calot's triangle indicated the location of the gallstones impacted in the CyD as a fluorescence defect (arrow). All images were obtained using LS1. See video, Supplementary Video 2, http://links.lww.com/MD/A308.

Accessory hepatic ducts^22^ were diagnosed by preoperative cholangiography in 10 patients. In 9 of these 10 patients, the accessory hepatic ducts were detected using FC after dissecting Calot's triangle (Figure 3A). FC after dissecting Calot's triangle also identified a thin hepatic duct running on the gallbladder bed (Figure 3B). With respect to anatomic variation in the CyD–CHD confluence, preoperative cholangiography identified the CyD running along the ventral or dorsal aspect of the CHD (spiral-type CyD–CHD junction)^8,23^ in 6 patients, and FC visualized all such variations prior to dissecting Calot's triangle (Figure 3C). FC allowed for visualization of gallstones in the CyD as defects in the fluorescence in 5 patients (Figure 3D), although no stones in the CBD were identified by FC (see Video, Supplementary Video 2, http://links.lww.com/MD/A308 [**Supplementary Video 2.** Fluorescence imaging of the accessory hepatic ducts, the variation of the cystic duct anatomy, and the cystic duct stones, 4 minutes 23 seconds, 7MB.], which demonstrates FC in patients with the bile duct anomalies). No adverse reactions to the ICG occurred.

### Clinical Factors Affecting the Ability of FC to Delineate the CyD–CHD Confluence

The effects of clinical factors on the ability of FC to delineate the CyD–CHD confluence are summarized in Table 3. Among the 6 patients with acute cholecystitis, FC before dissection of Calot's triangle enabled identification of the CyD–CHD confluence in 2 patients, whereas FC after dissection visualized the confluence in 5 of the 6 patients. The interval between intravenous injection of ICG and FC imaging before dissecting Calot's triangle was significantly longer in the patients in whom the CyD–CHD confluence could be visualized by FC than in those whom it could not be visualized (median [range], 90 [15–165] vs 47 [21–205] min, respectively; *P* < 0.01). The BMI had no predictive values for detection of the confluence by FC, before or after the dissection, in the present series.

**TABLE 3:**
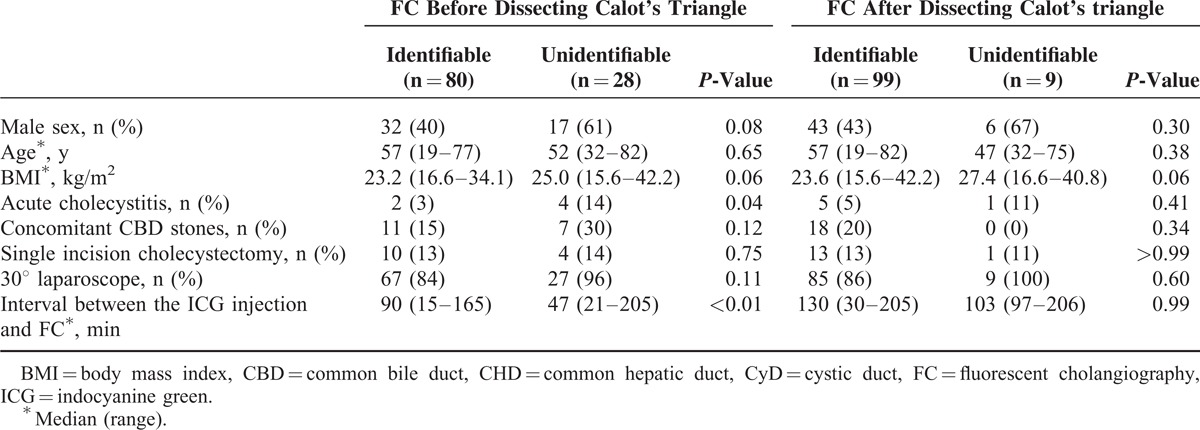
Clinical Factors Affecting the Ability of FC to Delineate the CyD–CHD Confluence (n = 108)

### Evaluation 2: Equipment-Related Factors Affecting Bile Duct Detectability by FC

**FIGURE 4:**
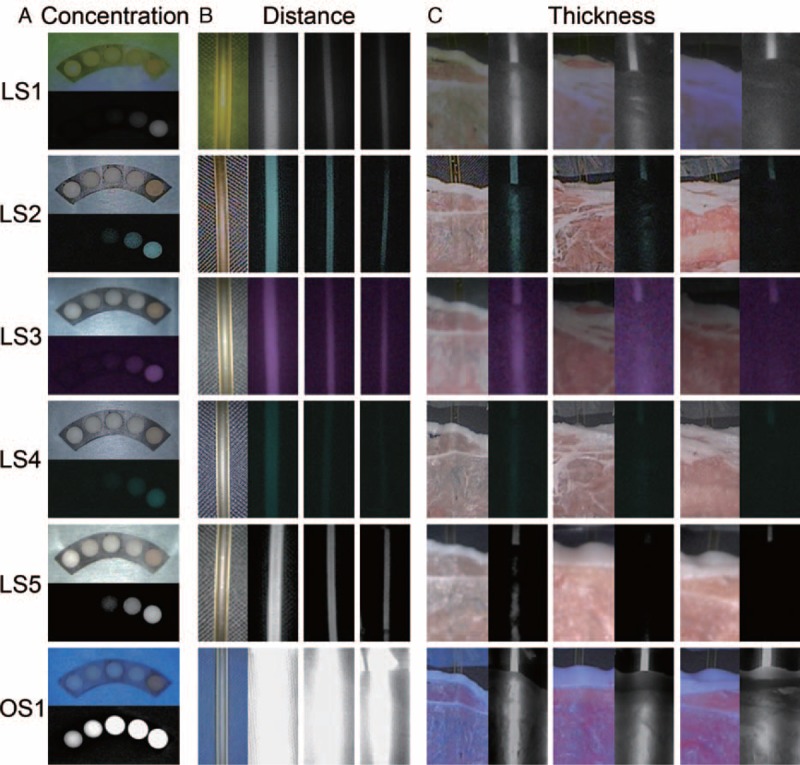
Features of fluorescence images and color images obtained by 5 laparoscopic imaging systems (LS1–5) and 1 open imaging system (OS1). (A) Color images (upper) and fluorescence images (lower) of a phantom containing 5 resins made of ICG diluted with alcohol to a dose of 0.5, 1.0, 2.0, 5.0, and 20.0 μmol/L (left to right). (B) Color images (leftmost) and fluorescence images (right) of the bile duct samples, which were obtained from 5, 10, and 15 cm above the samples (left to right). (C) Color images (left in each figure) and fluorescence images (right in each figure) of the bile duct samples, which are covered with 1, 3, and 5 slices of porcine tissues (left to right).

As shown in Figure 4, the features of the fluorescence and color images differed among the fluorescence imaging systems according to the wavelengths of excitation light and filters and the image processing used in each system.

**FIGURE 5:**
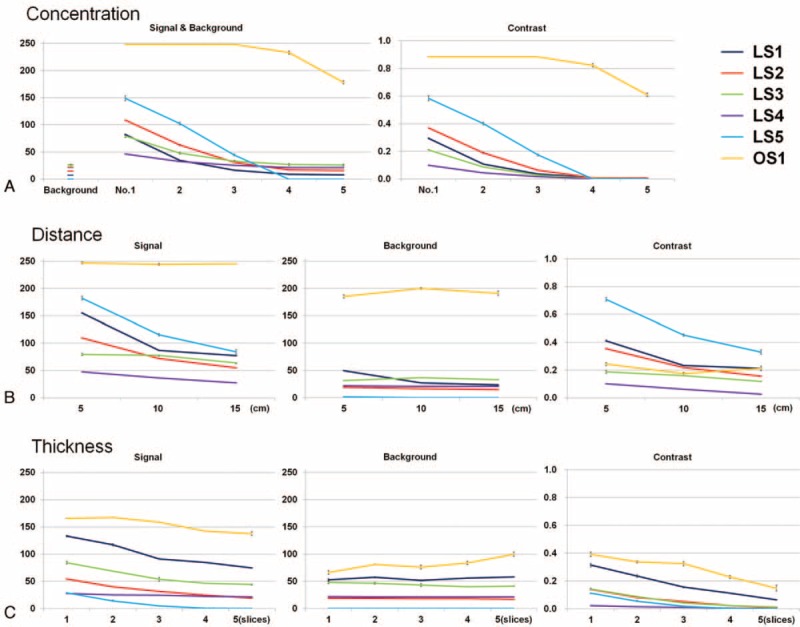
Signal and background FIs and signal contrast of the ex vivo samples obtained using 5 laparoscopic imaging systems (LS1–5) and 1 open imaging system (OS1). (A) FIs and signal contrast of resins obtained by 5 laparoscopic imaging systems were the highest in sample No. 1 (20 μmol/L of ICG) and then markedly decreased as the ICG concentration decreased. Conversely, the signal contrast obtained by OS1 remained above 0.6 even for sample No. 4 (0.5 μmol/L of ICG). (B) FIs and signal contrast obtained using the laparoscopic fluorescence imaging systems fell to approximately one-half when the distance from the bile duct samples and tip of the laparoscope increased from 5 to 15 cm. (C) FIs and signal contrast gradually declined as the thickness of the porcine tissues covering the bile duct samples increased.

Figure 5 demonstrates the FIs and signal contrast of the target regions on the fluorescence images obtained with 6 fluorescence imaging systems. With respect to the relationship between the signal contrast and ICG concentration, the resin made of ICG diluted to a dose of 20 μmol/L (0.16 mg/mL) led to the highest signal contrast for each laparoscopic imaging system. The signal contrast at this ICG concentration was significantly different among the laparoscopic systems used for fluorescence imaging, ranging from 0.10 ± 0.002 (mean ± SD) in LS4 to 0.58 ± 0.019 in LS5 (*P* < 0.01) (Figure 5A). The signal contrast obtained by the laparoscopic imaging systems decreased to <0.2 when ICG was diluted to ≤2.0 μmol/L, while that obtained by the open laparoscopic system (OS1) remained at >0.6 even for ICG diluted to 0.5 μmol/L.

The signal contrast obtained using the fluorescence imaging systems fell to approximately one-half when the distance from the bile duct samples and tip of the laparoscope increased from 5 to 15 cm (Figure 5B). In this setting, OS1 did not provide the highest signal contrast despite the highest FIs on fluorescence images of bile duct samples; this occurred because the FIs on the background were also high due to diffusion of the fluorescence signals around the sample.

The FIs and signal contrast gradually declined as the thickness of the porcine tissues covering the bile duct samples increased (Figure 5C). The FIs and signal contrast of LS5 in this series tended to be lower than those in the previous experiments without using porcine tissues, probably because the filter of this system was set steeply to cut off weak fluorescence signals penetrating the tissues.

## DISCUSSION

In the present series of LC, FC delineated the CyD–CHD confluence in two-thirds of the patients prior to dissection of Calot's triangle. This landmark anatomic structure of the extrahepatic bile ducts was identified by fluorescence imaging in 90% of patients by the end of dissection of Calot's triangle. Additionally, anatomic variations of the CyD and hepatic ducts, which are known risk factors for bile duct injury during cholecystectomy,^24^ were visualized by FC during the dissection procedures. For example, without the help of FC in the present series, it would have been difficult to understand the anatomy of the thin hepatic duct running on the gallbladder bed consistent with the original report in 1972.^25^ These results suggest that FC could be adopted as an effective navigation tool to delineate the bile duct anatomy during dissection of Calot's triangle until we reach the “critical view of safety.”

One of the key clinical factors in clear visualization of the CyD–CHD confluence by FC is the interval from the intravenous injection of ICG to the start of the imaging. In the present study, the median interval between the ICG injection and the initial FC was 100 min in patients in whom the CyD–CHD confluence could be identified by FC before dissecting Calot's triangle, which was approximately 40 min longer than that in patients who underwent incomplete FC. This is probably because adequate time is needed for the ICG excreted into the bile to move into the gallbladder through the CyD, although ICG is excreted into the bile within minutes after the intravenous injection.^18^ ICG should be administered before entry of the patient into the operation room to ensure better identification of the sequence of the CyD from the infundibular portion to the confluence with the CHD.

Another technical point for increasing detectability of the bile ducts by FC is full extension of Calot's triangle by lateral retraction of Hartmann's pouch of the gallbladder to reduce the thickness of the connective tissues around the bile ducts. It is also important to set the tip of the 0° or 30° laparoscope vertically to Calot's triangle to directly irradiate exciting light on the bile ducts directly and efficiently obtain fluorescence signals. The present ex vivo studies also indicated that the signal contrast on the fluorescence images could be increased by thinning the porcine tissues around the bile duct samples and decreasing the distance from the tip of the laparoscope.

The major limitation of FC is that it may fail to delineate the deeply located bile ducts during LC. Near-infrared light can penetrate human tissues only to a depth of about 5 to 10 mm, and the bile duct detectability of fluorescence imaging may be further decreased by the use of a laparoscopic imaging system compared with the use of FC with an open imaging system, especially around fat tissues, as suggested in the present ex vivo study. Thus, in patients with severe cholecystitis and/or obesity, FC may fail to elucidate the whole anatomy of the extrahepatic bile ducts buried in thick connective tissues prior to dissection of Calot's triangle. Under such adverse conditions, however, FC would still be useful because surgeons can perform FC easily and repeatedly while dissecting Calot's triangle to reduce the thickness of the connective tissues around the bile ducts, thereby avoiding bile duct injury and bile leakage from the bile duct stump.

The role of IOC during LC is not only to confirm the bile duct anatomy, but also to detect concomitant CBD stones.^26^ While FC is useful to detect gallstones impacted in the CyD, it would be difficult to visualize small CBD stones as defects on fluorescence images because of the presence of fluorescing bile around the stones. Conventional IOC and/or intraoperative ultrasonography should be added for patients with a likelihood of CBD stones, as well as when there remain concerns about the bile duct anatomy visualized by FC during dissection of Calot's triangle or prior to division of the CyD.

**FIGURE 6:**
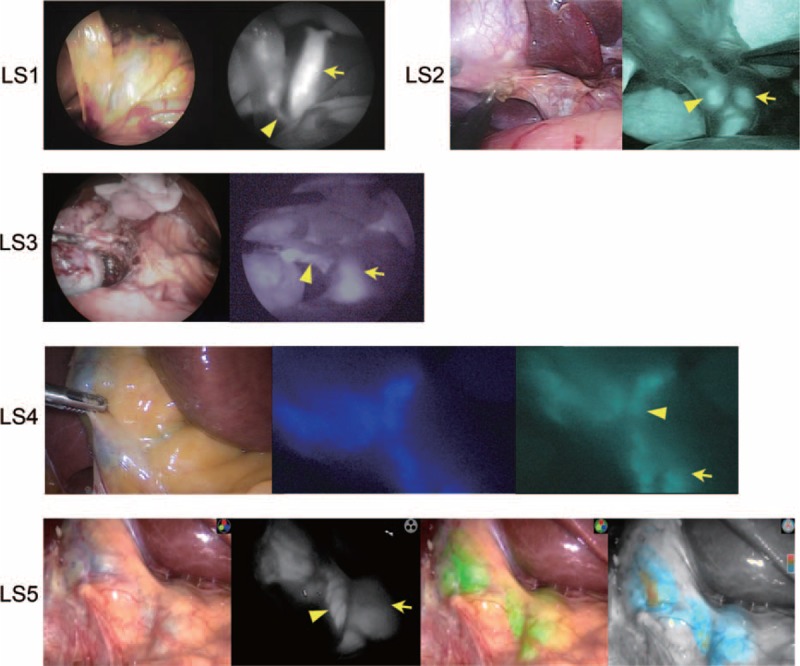
FC and corresponding color images of the extrahepatic bile ducts during LC obtained using five laparoscopic fluorescence imaging systems in the clinical setting. (LS1, LS2, LS3) Color images (left) and fluorescence images (right). (LS4) Color image (left), fluorescence image (middle), and fluorescence image with image processing similar to narrow-band imaging (spectra A mode, right). (LS5) Color image, monochromatic fluorescence image, pseudocolor fluorescence image (green) superimposed on color image, and pseudocolor fluorescence image (blue) on monochromatic background (from left to right). Arrows and arrowheads indicate the CHD and CyD, respectively.

Among the expanding applications of laparoscopic ICG-fluorescence imaging, such as identification of liver cancers,^27^ hepatic segments,^28^ lymph nodes,^29^ and blood perfusion,^30^ the most promising indication is cholangiography during LC. This is the most frequently performed surgical procedure worldwide; FC can utilize both the biliary excretion and fluorescence properties of ICG. Large prospective studies are currently being conducted to evaluate the efficacy and cost-efficiency of FC in enhancing the safety of LC. Prior to the performance of clinical trials, however, a fluorescence imaging system for FC should be optimized because, as demonstrated in the present study, the visualizability of fluorescence images and quality of color images are quite different depending on the imaging system used (Figure 6). Further technical innovations in the light source (optimization of intensity and wavelengths), image sensor (CCD/CMOS, resolution enhancement, and filter wavelengths), and image processing (superimpose imaging) are also needed to develop FC into an indispensable tool for bile duct navigation during LC.

In conclusion, FC is a simple navigation tool that is easy to use during LC. It can provide a road map of the extrahepatic bile ducts to reach the “critical view of safety” without any interventions involving the biliary tracts or exposure to radiation. Key factors for more accurate bile duct identification by FC are administration of ICG as far in advance as possible before surgery, sufficient extension of connective tissues around the bile ducts, and placement of the tip of the laparoscope close and vertically to Calot's triangle.
